# Metabolism via Arginase or Nitric Oxide Synthase: Two Competing Arginine Pathways in Macrophages

**DOI:** 10.3389/fimmu.2014.00532

**Published:** 2014-10-27

**Authors:** Meera Rath, Ingrid Müller, Pascale Kropf, Ellen I. Closs, Markus Munder

**Affiliations:** ^1^Department of Pharmacology, Institute of Medical Sciences, Faculty of Medical Sciences, Siksha ’O’ Anusandhan University, Bhubaneshwar, India; ^2^Section of Immunology, Department of Medicine, Imperial College London, London, UK; ^3^Department of Pharmacology, University Medical Center, Johannes Gutenberg University, Mainz, Germany; ^4^Third Department of Medicine (Hematology, Oncology, and Pneumology), University Medical Center, Johannes Gutenberg University, Mainz, Germany; ^5^Research Center for Immunotherapy, University Medical Center, Johannes Gutenberg University, Mainz, Germany

**Keywords:** macrophage, M1 and M2, arginine, arginase, nitric oxide synthase, immunoregulation, amino acid transporter

## Abstract

Macrophages play a major role in the immune system, both as antimicrobial effector cells and as immunoregulatory cells, which induce, suppress or modulate adaptive immune responses. These key aspects of macrophage biology are fundamentally driven by the phenotype of macrophage arginine metabolism that is prevalent in an evolving or ongoing immune response. M1 macrophages express the enzyme nitric oxide synthase, which metabolizes arginine to nitric oxide (NO) and citrulline. NO can be metabolized to further downstream reactive nitrogen species, while citrulline might be reused for efficient NO synthesis via the citrulline–NO cycle. M2 macrophages are characterized by expression of the enzyme arginase, which hydrolyzes arginine to ornithine and urea. The arginase pathway limits arginine availability for NO synthesis and ornithine itself can further feed into the important downstream pathways of polyamine and proline syntheses, which are important for cellular proliferation and tissue repair. M1 versus M2 polarization leads to opposing outcomes of inflammatory reactions, but depending on the context, M1 and M2 macrophages can be both pro- and anti-inflammatory. Notably, M1/M2 macrophage polarization can be driven by microbial infection or innate danger signals without any influence of adaptive immune cells, secondarily driving the T helper (Th)1/Th2 polarization of the evolving adaptive immune response. Since both arginine metabolic pathways cross-inhibit each other on the level of the respective arginine break-down products and Th1 and Th2 lymphocytes can drive or amplify macrophage M1/M2 dichotomy via cytokine activation, this forms the basis of a self-sustaining M1/M2 polarization of the whole immune response. Understanding the arginine metabolism of M1/M2 macrophage phenotypes is therefore central to find new possibilities to manipulate immune responses in infection, autoimmune diseases, chronic inflammatory conditions, and cancer.

## Introduction: Arginine in the Center of M1/M2 Macrophage Dichotomy

Macrophages are highly versatile cells, which are (i) crucial for infection control (“kill/fight mode”) and tissue homeostasis (“default mode”, phagocytosing cellular debris) and (ii) involved in disease pathophysiology in cancer, autoimmunity, metabolic, and fibrotic disorders (1). Macrophages react to a wide variety of external stimuli and are able to produce a multitude of effector molecules for intercellular communication, microbial defense, and modulation of inflammatory reactions (1). They induce, suppress, or modulate both innate and adaptive immune responses. Considering this enormous complexity it is reasonable to deduce classification schemes to create order and sense in the experimental results of macrophage research (2, 3). Potential macrophage diversity, both in terms of activation states, surface marker expression, metabolic phenotype, and interspecies differences clearly requires rigid standards for experimental set-up and reporting (4). While undue simplification hampers the comparability between studies (4), a reductionist approach tries to avoid getting lost in the complexities of macrophage biology and has both enormous power for the explanation of reality and can be the basis for experimentally testing of hypotheses. One should never forget that even the most sophisticated modern computers are based on the “0–1” dichotomy!

One of the most fruitful and reasonable classification of macrophages relates to their two main functions, namely, to kill/fight or to heal/fix. Within this classification view, macrophage biology is driven by two phenotypes (M1 for killing/fighting versus M2 for healing/fixing), which are also relevant in an evolving or ongoing immune response (5). M1 or M2 dominant macrophages then direct T lymphocytes to produce Th1 or Th2 responses, respectively, to further amplify M1 or M2 type responses in positive feed-back loops stabilizing the predominant immune phenotype in the respective setting of infection, tumor, or inflammation. The M1/M2 macrophage classification can be condensed into two opposing pathways for the metabolism of one amino acid: the preference of macrophages to metabolize arginine via nitric oxide synthase (NOS) to NO and citrulline or via arginase to ornithine and urea defines them as M1 (NOS) or M2 (arginase) macrophages (5). NOS or arginase are enzymes that catalyze a “reaction,” but we will use “pathway” here to illuminate that they are part of multi-enzyme pathways producing other physiologically important products.

In this introductory review, we will describe macrophage arginine metabolism, its functional consequences and how the macrophage arginine metabolic phenotype defines the two opposing M1 and M2 types of macrophages. While various molecules and features of macrophages are reciprocally or mutually exclusively associated with the M1 versus M2 phenotype, the dichotomous regulation of arginine metabolism is at the center of the different functions that are associated with M1 and M2 macrophages.

## Arginine: One Small Amino Acid for Macrophage Metabolism, a Giant Controller for Mammalian (Patho-)Physiology

Mammalian arginine metabolism is complex both at the level of the whole organism (6, 7) and at the level of the individual cell types (8) and we would like to adopt the term “argenomics” that was suggested by Sidney M. Morris Jr (7) for the regulation of gene expression via arginine availability in an even broader sense for the whole fascinating complexity of arginine-driven cellular regulation. Before focusing in on arginine metabolism of macrophages and its determining role for the M1/M2 dichotomy, let us first have a short overview on some historical facts relating to arginine in mammalian physiology. The story started nearly 130 years ago, arginine was first isolated in 1886 and was identified as a component of animal proteins in 1895 (8). The role of arginine in metabolic physiology was first demonstrated in 1932, when Krebs and Henseleit discovered the urea cycle. In 1981, Windmueller and Spaeth reported that the small intestine is the major source of citrulline for synthesis of arginine by the kidneys, now called the intestinal–renal axis for arginine synthesis on an organismal level (9). In 1987, it was shown that arginine is the precursor for macrophage citrulline and nitrite synthesis (10) and that arginine-derived NO is the elusive endothelium-derived relaxing factor (EDRF) (11, 12). Soon afterwards, NO was categorized as physiologically active intermediate of the arginine to nitrite (+nitrate) pathway in macrophages (13, 14) and endothelial cells (15). The discovery of the fundamental role of arginine-derived NO for human cardiovascular physiology already led to the award of the Nobel Prize in 1998 to Robert F. Furchgott, Louis J. Ignarro, and Ferid Murad. The importance of arginine has still risen since then, it is now clear that immune cell arginine metabolism is fundamentally involved in cancer, inflammation, infections, fibrotic diseases, pregnancy, and immune regulation in general (16–21). A huge responsibility for a small molecule!

## Macrophage Arginine Availability: Several Roads Lead to One Amino Acid

**Figure 1:**
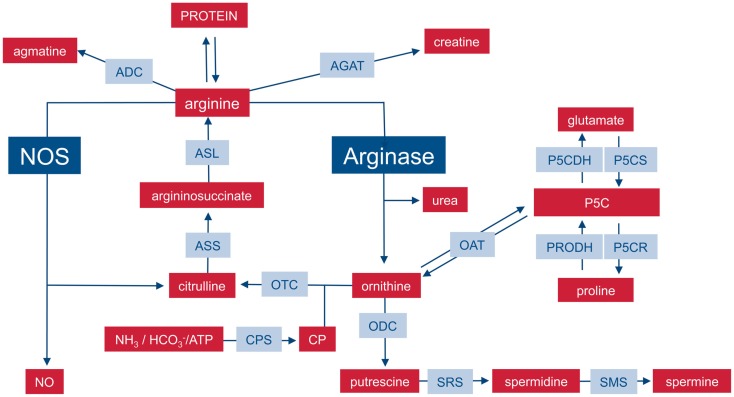
**Important pathways of mammalian arginine metabolism**. M1 and M2 macrophages are characterized by the metabolism of arginine via NOS or arginase with important functional consequences. This dichotomy is put into the context of other important pathways that can lead to the synthesis or degradation of arginine in mammalian cells. For sake of clarity, the diagram focuses on relevant enzymes (gray-shaded boxes), metabolites (red boxes), and the position of NOS and arginase within the network is highlighted. Various intermediate steps, by-products, or substrates are omitted and the reader is referred to more extensive chemical reviews (see text). ADC, arginine decarboxylase; AGAT, arginine:glycine amidinotransferase; ASL, argininosuccinate lyase, ASS, argininosuccinate synthase; CP, carbamoyl phosphate; CPS, CP synthase; NOS, nitric oxide synthase; OAT, ornithine aminotransferase; ODC, ornithine decarboxylase; OTC, ornithine transcarbamylase; P5C, pyrroline-5-carboxylate; P5CDH, P5C dehydrogenase; P5CR, P5C reductase; P5CS, P5C synthase; PRODH, proline dehydrogenase; SRS, spermidine synthase; SMS, spermine synthase.

On the level of the whole organism, arginine is a non-essential amino acid for healthy adult humans, but it has to be supplemented during growth or various disease states (6, 7) and has thus been characterized as a semi- or conditionally essential amino acid. Average arginine ingestion with a Western style diet is around 4–5 g/day and the normal plasma arginine level is 100–200 μM. Besides dietary intake, arginine is derived from cellular protein break-down or endogenous *de novo* arginine production. Mammalian arginine biosynthesis (Figure 1) involves mainly the amino acids glutamine, ornithine, and citrulline and the involved enzymatic steps are compartmentalized in different tissues and also on the subcellular level so that not all reactions can take place in each individual cell type or tissue. For a more detailed description of the chemical pathways of arginine metabolism, the reader is referred to the excellent review by Wu and Morris (8). (i) Glutamine can be converted to ornithine via glutaminase (yielding glutamate), pyrroline-5-carboxylate synthetase (P5CS), which is almost exclusively expressed in the intestinal mucosa, and ornithine aminotransferase (OAT). (ii) Ornithine transcarbamylase (OTC) and carbamoyl phosphate synthetase (CPS) are involved in the formation of citrulline from ornithine. The enzymes are restricted to the mitochondrial matrix of hepatocytes and epithelial cells of small and (to a minor extent) large intestine. This reaction is therefore a part of the hepatic urea cycle and also involved in intestinal synthesis of citrulline, which is released into the circulation. The proximal tubules of the kidneys take up most of the circulating citrulline, which is then converted within the kidney to arginine and again released into the circulation. (iii) Argininosuccinate synthetase (ASS) and argininosuccinate lyase (ASL) are cytosolic enzymes responsible for the biosynthesis of arginine from citrulline (and aspartate as a co-substrate). While ASS and ASL are expressed constitutively or inducibly in many different cell types, their degree of expression, and the efficiency of their catalytic pathways vary between different tissue types. On a whole body level, the latter enzymatic steps form the basis of the so-called intestinal–renal axis with intestinal production of citrulline (see above) and renal synthesis of arginine (7).

**Figure 2:**
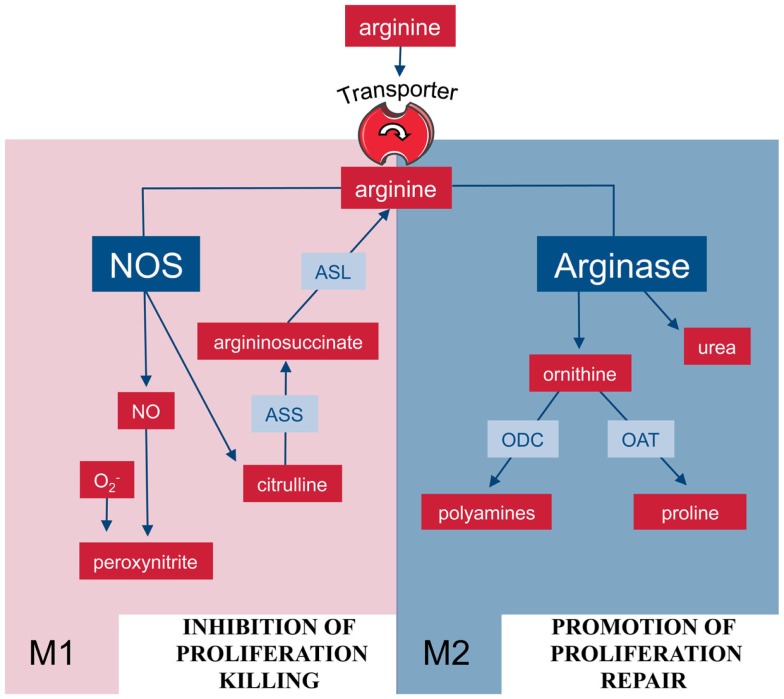
**Arginine metabolism via NOS or arginase is at the center of the M1/M2 polarization of macrophages**. M1 and M2 macrophages are characterized by the metabolism of arginine via NOS or arginase with important functional consequences. Abbreviations: see Figure 1.

M1 macrophages can also synthesize arginine in a cyclic fashion (Figure 2): during NO synthesis, arginine is converted to NO and citrulline via N^ω^-OH-arginine (22, 23). Murine macrophages have long been known to (i) upregulate ASS and constitutively express ASL when stimulated with the NOS-inducing agents lipopolysaccharide (LPS) and IFN-γ (24) and (ii) to partially rescue NO synthesis via citrulline uptake and ASS-mediated recycling to arginine (25). This set of reactions via ASS and ASL forms the so-called citrulline–NO cycle (26). The importance of this pathway for the resynthesis of arginine to ensure sufficient substrate supply for prolonged NO synthesis under arginine limitation has been recently demonstrated *in vivo* in murine mycobacteria infection (27). Despite the upregulation of ASS1, availability of arginine remains a rate-limiting step for synthesis of NO and cellular uptake of arginine also determines the amount of NO synthesized in case of NOS and ornithine in case of arginase (28, 29).

## Cellular Uptake of Arginine

The transmembranous arginine transport is one essential component of cellular arginine metabolism and important for the cells to perform their tasks. Amino acids do not pass membranous lipid bilayers freely, but are transported via specialized proteins. These amino acid transporters show different transport characteristics and specificities for the various amino acids, based on their physicochemical properties. Arginine, like the other cationic amino acids ornithine and lysine is preferentially taken up via members of the solute carrier family 7 (SLC7) (30–32). The subfamily of cationic amino acid transporters (CAT1–3, i.e., SLC7A1–3) recognizes exclusively cationic amino acids, while members of the subfamily of heteromeric amino acid transporters, y^+^LAT1 (SLC7A7) and y^+^LAT2 (SLC7A6), and b^0,+^AT transport also neutral amino acids. CAT and y^+^LAT proteins are widely distributed in various tissue types. CAT proteins are the main transporters for arginine uptake into cells, where the amino acid is then used for protein synthesis and all arginine-dependent metabolic pathways (7). CAT-1 is constitutively expressed in most tissues, with the exception of the liver, while CAT-2B is cytokine-inducible. CAT-2A is predominantly expressed in liver while CAT-3 is widely expressed during embryonal development but largely restricted to central nervous system and thymus in adults (30). In contrast to CAT, y^+^LAT proteins exchange primarily extracellular neutral against intracellular cationic amino acids and are therefore responsible for arginine export rather then import (30, 32). b^0,+^AT is expressed in epithelial cells of small intestine and kidney where it is responsible for the (re)absorption of cationic amino acids and cysteine (32). ATB^0,+^, a member of the SLC6 family, also transports both cationic and neutral amino acids and is expressed in the apical membrane of epithelial cells in various tissues (30).

The complex regulation of CAT expression has been studied quite extensively (30, 32, 33), whereas comparatively little is known about arginine transporter expression in most cells of the hematopoetic system in general or the immune system specifically. Induction of CAT-2 has been shown in murine macrophages upon both Th1 and Th2 cytokine stimulation (28), in murine dendritic cells (DCs) by retinoic acid (RA) (34) and in murine microglia upon stimulation with IFN-γ+/− LPS (35). In contrast, CAT-1 is either expressed constitutively (28) or even downregulated upon activation (35). Coordinated induction of CAT-2 and arginase (partially dependent on CAT-2 expression) has been demonstrated in macrophages during murine allergic lung inflammation, forming the basis of bleomycin-induced fibrosis (36) and also in RA-activated DCs (34). On the other hand, sustained production of NO in murine macrophages is also based on CAT-2 expression and CAT-2-mediated arginine uptake (37). The induction of CAT-2 in both M1 and M2 type murine macrophages has been shown to differ quantitatively between different mouse strains with important pathophysiological consequences: a deletion in the SLC7A2 promoter of C57BL/6 mice leads to impaired CAT-2 expression, reduced arginine uptake, and decreased susceptibility to *Leishmania* infection as compared to BALB/c mice (38). In contrast to murine macrophages, arginine transport is based on system y^+^L in IFN-γ-activated human primary monocytes (39) or LPS-stimulated alveolar macrophages (40), another example of interspecies differences, which are so prominent in various aspects of arginine metabolism in the immune system (17, 41, 42).

A crucial principle of immune cell signaling is the constitutive preexistence or the activation-induced assembly of multiprotein complexes that facilitate efficient transduction of stimuli. In murine myeloid cells, enhanced arginine import via CAT-2 is coupled to the induction of the arginine-catabolizing enzymes arginase I (28) and NOS (28, 34, 35, 43). It remains to be analyzed if (i) further similar higher-order structures or coordinated enzyme induction, combining arginine transporters with specific arginine-metabolizing proteins, can be found in macrophages and if (ii) differences in M1 and M2 type cells do exist. This is a valid hypothesis since amino acid transporters are not only final elements of distinct signal transduction pathways, which immune cells need for nutrient supply, but are also rather intricately involved in complex metabolic networks in which they influence further downstream signaling nodes and metabolic pathways (44). Interestingly, the ornithine-derived polyamine spermine enhances the expression of CAT-1 mRNA in human retinal pigment epithelial cells (45). This observation leads to an interesting question regarding macrophages: do M2 type macrophages increase their arginine transport capacity via endogenous arginase-mediated synthesis of polyamines, which would then further amplify arginase-based metabolism in a positive feed-back loop? An alternative scenario might result from the extracellular synthesis of ornithine after arginase has been secreted (46) or liberated unspecifically during cell death of myeloid cells (47). As ornithine is a cationic amino acid and substrate of cationic amino acid transporters, it (i) competitively inhibits arginine uptake and (ii) can be exchanged for intracellular arginine via CAT transporter proteins (31), potentially leading to a cellular depletion of arginine.

## Arginine Catabolism: A Bipolar World in Macrophages

In mammalian cells, arginine can be catabolized by four classes of enzymes (Figure 1): NOS, arginase, arginine decarboxylase (ADC), and arginine:glycine amidinotransferase (AGAT) (8). Although the enzymes are of course regulated and expressed in a cell-type-specific manner, the metabolism of arginine is potentially complex since its downstream metabolites encompass NO, urea, ornithine, citrulline, creatine, agmatine, glutamate, proline, and the family of polyamines (7, 8). In macrophages, arginine is a precursor for mainly two important metabolic pathways: it is metabolized either by inducible nitric oxide synthase (iNOS) to NO and citrulline or it is hydrolyzed by arginase to ornithine and urea (Figure 2). This fundamental dichotomy of macrophage arginine metabolism has wide ranging implications for their function as well as for the type and outcome of immune responses in which these innate immune cells are involved in. Before these consequences are discussed (see below), let us first look at the two important distinct arginine enzymatic pathways in macrophages in more detail.

### Nitric oxide synthase: Arginine – nitric oxide pathway

In 1980, Furchgott and co-workers discovered (i) the necessity of an intact endothelium for relaxation of isolated blood vessels and (ii) the presence of an endothelial cell-secreted unknown soluble relaxing factor (EDRF) (48). In 1987, this factor was found to be identical with NO (11) and arginine was revealed as the precursor for NO synthesis in cardiovascular physiology (15). In parallel studies, ${\mathrm{NO}}_{2}^{-}$ and ${\mathrm{NO}}_{3}^{-}$ were measured as end products of a novel oxidation pathway expressed in macrophages upon stimulation with LPS (49) or IFN-γ (50). Arginine was recognized as the biological precursor molecule of nitrite/nitrate released from activated macrophages (10). Further studies demonstrated that NO is an intermediate of macrophage arginine oxidation to the final end products nitrite/nitrate (14) and that NO synthesis is required for macrophage cytotoxic activity (22). In 1991, the enzyme converting arginine to NO was purified, cloned, and was named NOS (now known as neuronal NOS, nNOS) (51). Shortly after this, two additional NOS isozymes were cloned: iNOS from macrophages (52, 53) and endothelial NOS (eNOS) (54).

The three NOS isozymes differ in structure, distribution, regulation, and synthetic capacity, but they catalyze the same reaction: the incorporation of molecular oxygen and the release of NO from the terminal guanidino nitrogen group of arginine and generation of citrulline as a byproduct (22). All NOS enzymes are large homodimeric proteins with two functional domains: (i) an N-terminal oxygenase and catalytic domain, which binds an iron–protoporphyrin IX (heme) prosthetic group and the cofactor tetrahydrobiopterin (BH_4_) and (ii) a C-terminal reductase domain with binding sites for flavin mononucleotide (FMN) and flavin–adenine dinucleotide (FAD). The catalytic reaction involves two monooxygenation steps: (i) arginine is hydroxylated by O_2_ and NADPH to form N^ω^-hydroxy-l-arginine, which is then (ii) oxidized to yield NO and citrulline. All three NOS isoforms can also synthesize superoxide in the absence of arginine and BH_4_. This NOS-derived superoxide can react with NO to form peroxynitrite. Both nNOS and eNOS are constitutively expressed enzymes and calcium-dependent in their activity. In contrast, iNOS is regulated via inducible transcription and synthesizes NO independent of calcium since the essential subunit calmodulin is bound to iNOS even at low intracellular calcium concentrations. Most prominently known as microbicidal and inflammatory effector pathway in macrophages (22), iNOS activity has also been demonstrated in a variety of other cell types, e.g., hepatocytes (55), pulmonary epithelium (56), and colon epithelium (57). A variety of pro-inflammatory cytokines (e.g., IL-1β, IFN-γ, or TNF-α), microbial products (e.g., LPS), and hypoxia can induce macrophage iNOS transcription, whereas other cytokines (e.g., IL-4, IL-10, TGF-β) suppress iNOS gene transcription (58). Additive or synergistic activities of combinations of multiple cytokines are most efficient in inducing or suppressing iNOS gene expression. NO synthesis can also be limited by arginine availability and/or on the level of iNOS protein expression (59). NOS can further be inhibited by endogenously produced asymmetric dimethylarginine (ADMA) or pharmacologically by synthetic arginine analogs with substitutions at the terminal guanidino group, e.g., N^ω^-monomethyl-l-arginine (L-NMMA), N^ω^-nitro-l-arginine (L-NNA), or N^ω^-nitro-l-arginine methyl ester (L-NAME). Once iNOS has been translated, a prolonged production of potentially large amounts of NO is detectable. NO can act via stimulation of soluble guanylate cyclase to generate cyclic GMP within the target cell. Besides its physiological role as guanylate cyclase stimulator, NO is also a radical with a very short half-life of approximately 3–5 s and it reacts with a variety of molecules leading to (i) further reactive nitrogen species (RNS) like N_2_O_3_, peroxynitrite (ONOO^−^), or nitronium ion $\left({\mathrm{NO}}_{2}^{+}\right)$ in the presence of oxygen radicals (60) and (ii) nitrosylated proteins with potentially altered or impaired function.

### Arginase: Arginine – ornithine pathway

The enzyme arginase drives the second or alternative pathway of arginine metabolism in macrophages, catalyzing the hydrolysis of arginine to ornithine and urea. While arginase was known as an enzyme of the hepatic urea cycle since the discovery of the latter in 1932 by Krebs and Henseleit, it is also expressed in many non-hepatic cells. There are two isozymes of arginase (arginase I and arginase II), which catalyze the same biochemical reaction but differ in cellular expression, cell-type-specific regulation, and subcellular localization (17, 61). Hepatic urea cycle arginase I is expressed as a cytosolic enzyme, while human granulocyte arginase I is found in the granular compartment (41) and arginase II is a mitochondrial enzyme (61). It was initially demonstrated that murine macrophage arginase is inducible by PGE_2_ (62), Th2 cytokines, and cAMP, both alone (62) and synergistically acting together (63). The molecular details of this gene regulation were then clarified: the transcription factors STAT-6 and CEBP/β assemble at an enhancer element 3 kb upstream from the basal promoter of arginase I and Th2 cytokine-mediated murine arginase I mRNA induction is controlled by this mechanism (64). Meanwhile, human macrophage arginase I expression was demonstrated by synergistic induction with cAMP increasing treatments (PDE4 inhibition) in combination with IL-4 or TGF-β (65). The molecular details of this induction were also clarified recently in the murine RAW264.7 macrophage cell line, involving STAT-6 and CEBP/β binding to an IL-4 response element in the arginase I promoter (66). Another layer of complexity comes into play by the demonstration of pathogen-induced toll-like receptor (TLR)-mediated induction of arginase I in murine macrophages (67, 68).

Ornithine serves as a substrate for ornithine decarboxylase (ODC), a rate-limiting enzyme in the synthesis of polyamines. Polyamines are small, polycationic molecules, which regulate a multitude of cellular processes like DNA replication, protein translation, cell growth, and differentiation (69, 70). Much less is known about an involvement of polyamines in immune reactions: the polyamine spermine, e.g., inhibits pro-inflammatory cytokine synthesis of human, LPS-stimulated PBMC (71), arginine transport (72), and NO synthesis in rat (72) and murine macrophages (73). Ornithine, via the enzyme OAT, is also a precursor amino acid for the synthesis of proline, which itself is essential for the synthesis of collagen. Accordingly, arginase-derived ornithine might be important in tissue (re-)modeling processes. This hypothesis was supported by studies that demonstrated an increase in arginase levels in fibrotic lung disease (74) or allergic asthma (75, 76).

What are the biological functions of arginase-expressing macrophages? While there is ample evidence, at least in the murine system, of the fundamental role of NO-producing macrophages for infection control (22), a multitude of pathophysiological scenarios have also been described in which arginase-expressing macrophages are key players (17). In the initial groundbreaking analysis on macrophage phenotypes during wound healing, Mills and co-workers (77, 78) showed that arginase-expressing and ornithine-producing macrophages were crucial for wound healing as opposed to NO-producing macrophages, which dominated the initial phase of antibacterial inflammation. The same two macrophage phenotypes were then also correlated with tumor growth (arginase/ornithine) or tumor killing (NOS/NO) (79). Based on these dramatic differences in function, the two different macrophage populations were then named as M1 and M2, based on their route of arginine metabolism (Figure 2) (5). In general, M2 type macrophages act as anti-inflammatory cells (“healing” mode) via diversion of arginine away from NOS or via the synthesis of downstream products derived from the ornithine that is generated via arginase (see above). For illustration, we want to list just a few, more recently published examples for the role of macrophage or, in this context, also DC arginase in (i) infection-induced inflammation, (ii) immune evasion in tumor and infection, (iii) regulation of fibrosis, and (iv) direct control of parasite growth. (i) The anti-inflammatory property of macrophage arginase during infection was shown in murine schistosomiasis where excessive tissue injury is prevented by arginase-expressing macrophages due to suppression of pro-inflammatory cytokines IL-12 and IL-23 and the maintenance of the Treg/Th17 balance (80). (ii) IL-6-induced arginase 1 in DCs leads to downmodulation of MHC-II and this is subsequently responsible for suppression of CD4^+^ T cell-mediated antitumoral immunity (81). Immune evasion of chronic *Helicobacter pylori* infection is mediated by arginase II induction in gastric macrophages, due to inhibition of NOS-mediated bacterial killing and suppression of pro-inflammatory cytokine production (82). (iii) Macrophage arginase I restricts Th2 cytokine driven inflammation and fibrosis in murine schistosomiasis (83) although macrophage arginase itself can be pro-fibrogenic via direct production of proline as collagen precursor in schistosomiasis (84). (iv) Macrophage arginase-mediated synthesis of polyamines enhances growth of intracellular *Leishmania* parasites in murine macrophages (85) and arginase-expressing granulocytes and the levels of arginase activities are markers of disease severity in human visceral leishmaniasis and in HIV infections (86–88).

One further important consequence of macrophage arginase expression is reduction of extracellular arginine. This is most likely more apparent in the immediate microenvironment of M2 macrophages due to the continuous flux of nutrients and the arginine synthetic capacity of the whole organism. Suppression of T cell activation via arginine depletion has been studied quite extensively *in vitro* (47, 89–91) and is known as one of the prime immunosuppressive mechanisms of arginase-expressing myeloid-derived suppressor cells (MDSC) (18). In contrast, its role in macrophage-driven pathophysiology *in vivo* is still not really clear in most relevant disease entities and needs to be analyzed in the future. We speculate that M2/arginase macrophages might be more efficient in the induction of extracellular arginine depletion since there is no intracellular reconstitutive mechanism for arginine recycling for M2 macrophages as opposed to M1 macrophages, which can use the citrulline–NO rescue pathway. Suppression of T cell activation, proliferation, and/or differentiation by macrophage arginase I was shown *in vivo* in murine disease models dominated by M2 macrophages, like schistosomiasis (80, 83) or leishmaniasis (92). Interestingly, extracellular arginine depletion has also been shown to inhibit ERK1/2 activation and subsequently pro-inflammatory cytokine production in LPS-stimulated macrophages (93). It will be interesting to study a potential influence of intracellular arginine depletion on potential pro-inflammatory signaling pathways within macrophages and to analyze if there is regulation of innate immune responses and macrophage polarization already at such basic level. Clearly, arginine depletion does not inhibit immune responses broadly and indiscriminately: important activation aspects of T cells (91, 94) and granulocytes (95) are preserved in an arginine-depleted milieu and other cellular responses, e.g., induction of arginine transport protein CAT-1 (33), are even enhanced in eukaryotic cells under arginine nutrient deprivation.

### Interactions between NOS and arginase pathways

Nitric oxide synthase and arginase can both antagonize or synergize in the generation of oxidative and nitrosative stress: inadequate supply of arginine (or the cofactor tetrahydrobiopterin) leads to the production of superoxide $\left(O_{2}^{-}\right)$ instead of NO, increasing oxidative stress and also the production of peroxynitrite (22). In general, though, mutually exclusive expression of iNOS and arginase I in individual macrophages prevails (96) and there are multiple cross-inhibitory interactions between the two arginine metabolic pathways in macrophages: NOHA, the intermediate product in NO synthesis, is a potent inhibitor of both arginase isoforms (97). In non-macrophage cell types, it was also demonstrated that NO is an effective inhibitor of ODC via nitrosylation of the enzyme with consecutively reduced polyamine synthesis (98). Arginase can limit arginine availability for NO synthesis, as demonstrated by pharmacological arginase inhibition in different types of macrophages (99). The expression of iNOS is translationally controlled by the availability of arginine (100) and in murine M2 macrophages, induced by the cytokine IL-13, iNOS translation, and NO production are restricted by arginase-mediated arginine depletion (59). Polyamines and/or polyamine aldehyde metabolites can inhibit NO synthesis in murine and rat macrophages (101). Spermine suppresses the induction of both iNOS and CAT-2B arginine transporter (72) and inhibition of ODC-mediated polyamine synthesis in murine macrophages enhances LPS-induced iNOS expression and NO synthesis (102). Spermine also inhibits *H. pylori*-induced iNOS protein translation in the RAW264.7 macrophage cell line and siRNA-mediated ODC inhibition enhances macrophage NO-mediated bacterial killing (103).

In summary, these biochemical crossregulatory interactions are in line with the concept of two types of polarized macrophages – M1/NOS versus M2/arginase – which are defined not only by the intracellular fate of arginine, but – most importantly – also by its associated functional consequences.

## M1/NOS Versus M2/Arginase Macrophages: Novel Aspects

The earlier simplified scheme of “proinflammation = M1/Th1” versus “anti-inflammation = M2/Th2” has meanwhile been clarified as one possible scenario of a more broader conceptual framework: M1 versus M2 polarization clearly leads to opposing outcomes of inflammatory reactions, but depending on the inflammatory or infectious context, M1 and M2 macrophages can be central players of both pro- or anti-inflammatory reactions. Notably, M1/M2 macrophage polarization can be driven by microbial infection or innate danger signals without any influence of adaptive immune cells, secondarily driving the Th1/Th2 polarization of the evolving adaptive immune response (104). Microbial stimulation of macrophages via TLRs leads to the activation of certain transcription factors (e.g., NF-κB, AP-1), which upregulate pro-inflammatory cytokines like IFN-γ and TNF-α leading to M1 macrophage polarization with high iNOS expression whereas cytokines like IL-4 or IL-13 lead to STAT6 phosphorylation with consecutive arginase expression and varying further aspects of M2 polarization (mannose receptor, Ym1, Fizz1).

Various novel aspects regarding the evolution of the M1/M2 macrophage polarization in light of the differential expression of NOS versus arginase have been clarified recently and some examples are summarized in the following sections:

### Novel exogenous environmental factors influencing the NOS/arginase balance

Tumor cells are known to metabolize glucose preferentially via aerobic glycolysis, known as “Warburg phenomenon,” and this leads to high concentrations of lactate in the tumor microenvironment. This tumor cell-derived lactic acid is a potent inducer of arginase I expression in tumor-associated macrophages (TAM) and these M2/arginase macrophages then foster tumor growth (105). Since activated T cells also use aerobic glycolysis with consecutive production of lactic acid, it will be interesting to study if the same mechanism of arginase I induction is also operative in macrophage-T cell interactions. Gliadin, a major component of cereal gluten, and therefore omnipresent in our daily food, was shown to induce arginase I in human monocytes (106). A parallel stimulation with IFN-γ leads to a reduction of cellular arginine efflux via downregulation of the arginine export protein y^+^LAT-2 (SLC7A6), thereby increasing intracellular availability of arginine for gliadin-induced arginase (106). Finally, growth factors have also entered the M1/M2 macrophage world: in a hamster model of visceral leishmaniasis, macrophage arginase I expression is driven by fibroblast growth factor-2 (FGF-2) and insulin-like growth factor-1 (IGF-1), which both signal via STAT6 and are amplified by co-stimulation with IL-4 (107).

### Intracellular signaling modules involved in M1(NOS)/M2(arginase) polarization

The tyrosine phosphatase Shp2 restricts M2 macrophage polarization as demonstrated by the preferential polarization of Shp2^−/−^ macrophages into an M2 direction with enhanced arginase expression, associated with a better protection against schistosomiasis (108). Also, PI3K/PTEN activity is involved in regulating arginase expression in murine macrophages since deletion of PTEN leads to M2 polarization via C/EBPβ and STAT3 (109). These results are in line with earlier reports demonstrating that SHIP phosphatase (which – like PTEN – also downregulates the PI3K pathway) dampens M2 polarization and arginase I expression in different types of tissue macrophages (110). Deacetylation of C/EBPβ is required for its binding to a DNA enhancer element and its role in IL-4-mediated arginase I induction in bone marrow-derived murine macrophages (111).

### Auto- or paracrine M1/M2 polarization

In murine macrophages, induction of arginase I by mycobacteria is driven by an autocrine–paracrine signaling loop: TLR-MyD88 mediate induction of the cytokines IL-6, G-CSF, and IL-10, which then induce arginase I, involving the transcription factors STAT3 and C/EBPβ (112). Respiratory syncytial virus (RSV) infection induces production of IL-4 and IL-13 of macrophages themselves and this leads to auto- and paracrine induction of arginase I expression in macrophages (68). It has long been known that murine DCs share the fundamental arginase/iNOS polarization with macrophages (113). A novel aspect here is that DCs produce RA, which then induces arginase I and the arginine transporter CAT-2B in the DCs themselves (34). This RA-mediated autocrine–paracrine induction of arginase I in DCs is induced by binding of RA to an RA-responsive element in the 5′ non-coding region of the arginase I gene and is enhanced by known exogenous arginase inducers like IL-4 or GM-CSF (34).

### M1/M2 polarization *in vivo*

In human tuberculosis, the distribution of M1 (iNOS) and M2 (arginase) macrophages is spatially organized within granulomas: M1 macrophages can preferentially be found in the inner region closer to viable mycobacteria, whereas a higher frequency of M2 macrophages is detectable on the outer “healing” margins. This clearly forms an organized microenvironment in which antibacterial (M1) responses are physically separated by M2-based anti-inflammation and fibrosis from uninvolved tissue (96). In a murine tuberculosis model, overexpression of IL-13 precipitates expansion of the M2 arginase-expressing macrophage response to the pathogens recapitulating human pathology of post-primary tuberculosis, while the endogenous inhibition of arginase I expression via NOHA restrains arginase expression and pathology (114). This is in line with an earlier study showing a disease-exacerbating role of macrophage arginase (67). In contrast to the latter two reports, a recently published study analyzed the role of macrophage arginase in a hypoxic model of *Mycobacterium tuberculosis* granuloma formation, in which iNOS-based synthesis of RNS is impaired and which likely reflects *in vivo* reality. Here, it was shown that arginase I expression in granuloma-associated macrophages restricts immune pathology since macrophage-specific deletion of arginase I led to larger granulomas and bacterial burden load (115). These discrepant study results clearly demonstrate the fundamental importance of the microenvironment and the multitude of potential factors that act on macrophages *in vivo* and which can simply not be mimicked *in vitro*.

## Summary and Outlook

The concept of macrophage M1/M2 dichotomy, based on differential usage of arginine via NOS or arginase was born 25 years ago based on analyses of healing wounds (5, 104). Since then, we have witnessed a bewildering explosion of knowledge regarding macrophage surface markers, activation requirements, signal transduction elements and gene regulation. Within this universe of complexity the simple discriminator arginase versus NOS expression has not only remained valid to explain how a mammalian organism deals with wounding (which is important enough in itself) but also has demonstrated enormous power to better explain and understand such diverse problems as cancer control versus cancer-induced immunosuppression, autoimmune pathology versus preservation of tolerance to self, infection control versus chronicity or death due to infection as well as tissue healing versus exaggerated fibrosis. Despite this scientific progress during the last quarter-century, we have by far not reached the summit of the Everest, but rather a solid base camp I, in which to plan and prepare the next steps. The arginase/iNOS dichotomy of macrophage amino acid metabolism has counterparts in the other major components of cellular metabolism: (a) genes of fatty acid oxidation are preferentially expressed in M2 macrophages and inhibition of fatty acid oxidation leads to an abrogation of M2 activation (116); (b) M1 macrophages preferentially use glycolysis and glutamine anaplerosis while M2 macrophages preferentially use oxidative metabolism (117). The emerging interconnections between macrophage metabolism and M1/M2 polarization (118) have recently been reviewed (119, 120) and we anticipate exciting progress in this field in the upcoming years. Another crucial aspect of the M1/M2 dichotomy that eagerly awaits more progress and clarification is the current discrepancy between murine and human macrophage biology in terms of iNOS/arginase expression and regulation (17, 42, 121). We definitely need more carefully executed *in vivo* and *ex vivo* analyses of human macrophage activation phenotypes in diverse disease settings. This will then also lay the foundation for targeted therapeutic intervention to harness the enormous power of the arginine metabolic phenotypes of M1/M2 macrophage polarization.

## Conflict of Interest Statement

The authors declare that the research was conducted in the absence of any commercial or financial relationships that could be construed as a potential conflict of interest.
